# Stillbirths and very low Apgar scores among vaginal births in a tertiary hospital in Ghana: a retrospective cross-sectional analysis

**DOI:** 10.1186/1471-2393-14-289

**Published:** 2014-08-28

**Authors:** Edward Tieru Dassah, Alexander Tawiah Odoi, Baafuor Kofi Opoku

**Affiliations:** Department of Obstetrics and Gynaecology, Komfo Anokye Teaching Hospital, Kumasi, Ghana; School of Medical Sciences, Kwame Nkrumah University of Science and Technology, Kumasi, Ghana

**Keywords:** Stillbirth, Apgar score, Vaginal delivery, Ghana

## Abstract

**Background:**

Data pertaining to risk factors associated with stillbirths and very low Apgar scores is very sparse. This study was conducted to determine the prevalence of, and examine the socio-demographic and obstetric factors associated with stillbirths and very low Apgar scores among vaginal births in a tertiary health facility, Ghana.

**Methods:**

A retrospective cross-sectional review of vaginal deliveries conducted at a teaching hospital in Ghana from 1^st^ January to 31^st^ December, 2009. Background characteristics and obstetric history of the mother as well as the vital status of the baby at birth were extracted. Risk factors associated with stillbirths and very low Apgar scores were examined using binomial regression with a log-link function, and population attributable fractions calculated for significant risk factors.

**Results:**

Of the 8,758 deliveries which met the inclusion criteria, 5.9% of the babies were stillbirths, and 6.5% and 1.9% of live births had very low Apgar scores in the first and fifth minutes respectively. Preterm delivery, hypertensive disorders in pregnancy, breech delivery and vacuum extraction were significant risk factors for stillbirths and very low Apgar scores in the fifth minute of life. Low birth weight was also a significant risk factor for very low Apgar scores.

**Conclusion:**

The prevalence of stillbirths and very low Apgar scores were high. Improving the quality of obstetric care during labour and delivery may help improve these adverse vaginal birth outcomes.

## Background

Over 3 million stillbirths occur globally each year, nearly all of which are in low-income countries [1–4]. Stillbirth rates are particularly high in sub-Saharan Africa, where up to 14% of deliveries could result in stillbirths. These rates are relatively higher in West Africa [2–7]. Apgar scores are used to assess the condition of the newborn in the first critical minutes of life and can be useful in evaluating neonatal resuscitation and survival [8, 9], especially in low resource settings. About 8-38% of babies delivered in West Africa may have low Apgar scores. Over two-thirds of babies with very low Apgar scores may result in perinatal mortality [6, 10, 11]. Stillbirths and Apgar scores are important indicators of the quality of obstetric care [6, 7, 10, 11]. Non-infectious maternal and obstetric risk factors for stillbirths that are likely to be important in sub-Saharan Africa include socio-demographic and reproductive factors, hypertensive states in pregnancy, gestational age at delivery, birth weight and type of vaginal delivery [4–7, 10, 12]. For example, preterm births and low birth weight infants are associated with increased risks of stillbirths, and nearly a fifth of such babies are stillborn [5].

The vaginal route remains the commonest mode of delivery in all health care facilities. A few studies have reported the prevalence of adverse birth outcomes and the maternal and reproductive factors associated with these outcomes in sub-Saharan Africa [4–7, 10, 13]. However, data regarding the contribution of socio-demographic and obstetric risk factors to stillbirths and very low Apgar scores of babies delivered vaginally remains very sparse. Increasing attention for these factors and focusing on interventions to reduce adverse vaginal birth outcomes will contribute immensely towards the attainment of Millennium Development Goal 4 on under-five mortality.

The main aim of this study was to determine the prevalence of stillbirths and very low Apgar scores among vaginal births in a tertiary health facility in Ghana, and examine the socio-demographic and obstetric factors associated with these adverse vaginal birth outcomes.

## Methods

This is a retrospective cross-sectional study of vaginal deliveries conducted at Komfo Anokye Teaching Hospital (KATH), a tertiary referral facility in Kumasi, Ghana from 1^st^ January to 31^st^ December, 2009. The hospital receives referrals from at least five out of the 10 regions in the country. About 13,000 deliveries are conducted in the hospital annually and nearly three-quarters of these are vaginal deliveries. There are three labour wards in the hospital, one of which is located in the intensive care unit for hypertensive disorders in pregnancy. Women admitted to these labour wards are monitored at regular intervals for maternal and fetal conditions as well as the progress of labour using the modified WHO partograph [14]. Intermittent auscultation of fetal heart tones is usually done at 30 and 5 minute intervals during the first and second stages of labour respectively using the fetal (Pinard) stethoscope; external electronic fetal monitoring with Doppler was not available in the delivery units at the time. Women whose cervical dilation is at least 8 cm on admission or those who require immediate delivery are managed without recourse to the partograph. The Department also has strict protocols for assisted vaginal deliveries. For example, the prerequisites for vaginal breech deliveries include extended or flexed breech, flexed fetal head, an estimated fetal weight not exceeding 3.5 kg, no previous uterine scar and an adequate maternal pelvis. The delivery must also be conducted by an experienced midwife or doctor [15, 16].

The hospital keeps a delivery register of all deliveries conducted in its labour wards. All records in the register were reviewed. All women who delivered vaginally at KATH within the study period were eligible for inclusion. Births without gestational age and birth weights were excluded. For each birth record, we abstracted information on the date of delivery, socio-demographic characteristics of the mother, obstetric factors such as parity, presentation, number of foetuses (single or multiple pregnancy), hypertensive disorders in pregnancy (comprising chronic hypertension, pregnancy-induced hypertension, pre-eclampsia and eclampsia) and the type of vaginal delivery (spontaneous vertex, breech or vacuum delivery). The sex, birth weight and Apgar scores of each baby at delivery were also extracted.

The main outcomes examined in this study were stillbirth and very low Apgar scores. Stillbirth was defined as death of a fetus weighing at least 1000 g or after 28 completed weeks of gestation occurring before the complete expulsion or extraction from its mother in accordance with World Health Organisation-agreed definition of stillbirth for international comparison [17]. A viable fetus was considered stillborn if it did not show any evidence of life at birth such as breathing, beating of the heart, pulsation of the umbilical cord or definite movement of voluntary muscles, whether or not the umbilical cord had been cut or the placenta was attached [17]. Apgar scores were assigned by the usual criteria at one and five minutes after delivery [8, 9]; scores of 0–3 were considered very low [9]. Preterm deliveries were births of infants occurring after 28 completed weeks but before 37 completed weeks of gestation. Low birth weight babies were infants born after 28 completed weeks of gestation and weighing less than 2500 g at birth. Gestational age was the number of days between the first day of the last menstrual period and the date of delivery expressed in completed weeks using ultrasound or clinical assessment as documented in the delivery record. Parity was the number of previous pregnancies carried beyond 28 completed weeks of gestation irrespective of the outcome.

Data was double entered into Microsoft Excel database, cleaned and transferred to Stata 11.0 (StataCorp, Texas, USA) for statistical analysis. Categorical variables were compared using χ^2^ test while continuous variables were compared using student t-tests. To quantify the risk of stillbirth or very low Apgar scores with the maternal and obstetric factors, crude and adjusted relative risks (RRs) with corresponding 95% confidence intervals (CIs) were calculated using univariable and multivariable binomial regression with a log-link function. Univariable analysis was performed to examine the association of each risk factor with stillbirth or very low Apgar scores and factors which reached statistical significance at P < 0.1 were entered into the multivariable model using the forward stepwise approach. Factors which remained significant (P < 0.1) were included the final model for each outcome. Excluded risk factors were retested in the final model one at a time to confirm lack of association. Statistical significance was assessed using the likelihood ratio and Wald tests. Tests for linear trend were performed for variables with more than two ordered categories.

The study was approved by the Committee on Human Research, Publications and Ethics of the Kwame Nkrumah University of Science and Technology, Kumasi, Ghana. This research adhered to the STROBE guidelines for observational studies.

## Results

A total of 12,190 deliveries were conducted in the period under study: 8,856 (72.6%) were vaginal deliveries, out of which 8,758 (98.9%) records met the inclusion criteria. Ninety-eight (1.1%) vaginal birth records were excluded for the following reasons; 67 (0.76%) babies weighed less than 1000 g at birth, 26 (0.29%) were born before 28 completed weeks of gestation and 5 (0.06%) lacked information on the birth weight and estimated gestational age at delivery.

The demographic and reproductive characteristics of the women are shown in Table 1. The mean age was 27.4 years (standard deviation 6.1), with a range of 16–42 years. The median parity was 1 (interquartile range 0–2). Most (82%) of the women were in employment. Four hundred and sixty-one women (5.3%) had hypertensive disorders in pregnancy. Two hundred and ninety-seven (3.4%) and 106 (1.2%) of the deliveries were breech and vacuum-assisted respectively. There were more male babies compared to females at birth (52% vs. 48%). These characteristics were similar among women with live births except that 4.7% and 2.7% of women who delivered their babies alive had hypertensive disorders of pregnancy and breech deliveries respectively.

**Table 1:** **Background characteristics and obstetric outcomes**

	Live births only ^a^ N = 8,234 n (%)	Total number of deliveries ^b^ N = 8,758 n (%)
Age group (years)		
12-19	800 (9.7)	841 (9.6)
20-35	6,493 (79.0)	6,899 (78.9)
36-50	927 (11.3)	1,003 (11.5)
Occupation		
Skilled	780 (9.8)	809 (9.6)
Semi-skilled	5,734 (72.0)	6,113 (72.2)
Unemployed	1,452 (18.2)	1,543 (18.2)
Religion		
Christian	6,750 (84.2)	7,164 (84.1)
Moslem/other	1,263 (15.8)	1,357 (15.9)
Parity		
0	2,877 (36.0)	3,056 (35.9)
1-4	4,634 (57.9)	4,915 (57.8)
5+	486 (6.1)	538 (6.3)
Number of singleton/multiple births		
Singleton (1)	7,955 (96.6)	8,464 (96.6)
Multiple (2 or 3)	279 (3.4)	294 (3.4)
Hypertensive disorder in pregnancy		
No	7,843 (95.3)	8,297 (94.7)
Yes	391 (4.7)	461 (5.3)
Type of delivery		
Spontaneous vaginal delivery	7,916 (96.2)	8,355 (95.4)
Breech delivery	224 (2.7)	297 (3.4)
Vacuum extraction	94 (1.1)	106 (1.2)
Condition of perineum after delivery		
Intact	5,771 (72.9)	6,171(73.5)
Episiotomy	579 (7.3)	609 (7.3)
Perineal tear	1,563 (19.8)	1,618 (19.2)
Sex of baby		
Female	3,989 (48.5)	4,223 (48.3)
Male	4,230 (51.5)	4,517 (51.7)
Birth weight		
Normal birth weight (≥2500 g)	7,307 (89.3)	7,597 (87.4)
Low birth weight (<2500 g)	876 (10.7)	1,099 (12.6)
Outcome of delivery		
Stillbirth	N/A	517 (5.9)
Live birth	8,234 (100)	8,234 (94.1)

Missing values among live births only^a^ and total number of deliveries^b^: 14 (0.17%)^a^ and 15 (0.17%)^b^ for maternal age; 268 (3.3)^a^ and 293 (3.4%)^b^ for occupation; 221 (2.7%)^a^ and 237 (2.7%)^b^ for religion; 237 (2.9%)^a^ and 249 (2.8%)^b^ for parity; 321 (3.9)^a^ and 360 (4.1%)^b^ for condition of perineum; 15 (0.18%)^a^ and 18 (0.21%)^b^ for sex of baby; 2 (0.02%)^b^ for gestation at delivery; 51 (0.6%)^a^ and 62 (0.7%)^b^ for birth weight; 7 (0.08)^b^ for outcome of delivery. N/A- not applicable.

### Stillbirth

Overall, 517 (5.9%) women had stillbirths. About one quarter of preterm or breech deliveries and 11% of vacuum extractions were stillbirths. On univariable analysis, stillbirth was associated with older mothers (>35 years), being unemployed or in semiskilled employment, grand multiparity (parity ≥ 5), hypertensive disorders in pregnancy, breech delivery, vacuum extraction and preterm births. Table 2 shows the crude and adjusted RRs of demographic and reproductive factors associated with stillbirth.

**Table 2:** **Factors associated with stillbirths**

	Stillbirths n (%)	Crude RR (95% CI)	Adjusted RR ^f^ (95% CI)
Maternal age group (years)^a^		P = 0.05; P trend = 0.02	P = 0.41; P trend = 0.19
12-19	41 (4.9)	0.84 (0.61, 1.16)	0.79 (0.55, 1.12)
20-35	400 (5.8)	1	1
36-50	75 (7.5)	1.29 (1.01, 1.65)	1.03 (0.76, 1.39)
Occupation^b^		P = 0.02	P = 0.07
Skilled	29 (3.5)	1	1
Semi-skilled	374 (6.1)	1.71 (1.17, 2.49)	1.56 (1.06, 2.30)
Unemployed	89 (5.8)	1.61 (1.06, 2.45)	1.59 (1.05, 2.43)
Parity^c^		P = 0.002; P trend = 0.06	P = 0.04; P trend = 0.23
0	178 (5.8)	1.03 (0.86, 1.25)	1.02 (0.83, 1.26)
1-4	277 (5.6)	1	1
5+	52 (9.7)	1.71 (1.27, 2.30)	1.56 (1.11, 2.18)
Type of delivery		P < 0.001	P < 0.001
Spontaneous vaginal delivery	433 (5.2)	1	1
Breech delivery	72 (24.3)	4.69 (3.65, 6.02)	2.70 (2.04, 3.57)
Vacuum extraction	12 (11.3)	2.18 (1.23, 3.87)	2.67 (1.50, 4.76)
Number of singleton/multiple births		P = 0.56	
Singleton	502 (5.9)	1	-
Multiple	15 (5.1)	0.86 (0.51, 1.44)	-
Hypertensive disorder in pregnancy		P < 0.001	P < 0.001
No	453 (5.5)	1	1
Yes	64 (14.1)	2.58 (1.98, 3.35)	1.77 (1.32, 2.36)
Sex of baby^d^		P = 0.14	-
Female	232 (5.5)	1	-
Male	283 (6.3)	1.14 (0.96, 1.36)	-
Gestation at delivery^e^		P < 0.001	P < 0.001
Term	359 (4.4)	1	1
Preterm	157 (26.9)	6.13 (5.08, 7.39)	5.15 (4.18, 6.35)

^a^1 (0.2%) missing value for age; ^b^25 (4.8%) missing values for occupation; ^c^10 (1.9%) missing values for parity; ^d^2 (0.4%) missing values for sex of baby; ^e^1 (0.2%) missing values for gestation at delivery; ^f^Adjusted for all other variables in the table except number of singleton/multiple births and sex of baby; P-values are based on the Wald test; RR-Relative risk; CI-Confidence interval.

On multivariable analysis, stillbirths were significantly higher among grand multiparous women (aRR, 1.56; 95% CI, 1.11-2.18), breech delivery (aRR, 2.70; 95% CI, 2.04-3.57), vacuum extraction (aRR, 2.67; 95% CI, 1.50-4.76), hypertensive disorders in pregnancy (aRR, 1.77; 95% CI, 1.32-2.36) and preterm delivery (aRR, 5.15; 95% CI, 4.18-6.35). Adolescent mothers were less likely to have stillbirths compared to older mothers, although this was not statistically significant.

### Very low Apgar scores at 1 and 5 minutes

Five hundred and twenty-four (6.5%) of 8,125 live births for whom Apgar scores were documented in the first minute of life had very low Apgar scores (0–3). The median Apgar score in the first minute was 6 (interquartile range 5–7). Nulliparity (aRR, 1.46; 95% CI, 1.20-1.77), preterm birth (aRR, 2.62; 95% CI, 1.94-3.54), breech delivery (aRR, 2.44; 95% CI, 1.76-3.37), vacuum extraction (aRR, 2.61; 95% CI, 1.46-4.64), low birth weight (aRR, 2.18; 95% CI, 1.67-2.85) and male sex (aRR, 1.30; 95% CI, 1.09-1.56) were predictive of very low Apgar scores in the first minute of life. The risk of having very low Apgar scores in the first minute increased with decreasing parity (P trend < 0.001), see Table 3.

**Table 3:** **Obstetric factors predictive of very low Apgar scores at 1 minute and 5 minutes among live births**

	Very low Apgar scores (0–3) at 1 minute ^a^			Very low Apgar scores (0–3) at 5 minutes ^b^		
**Obstetric factor**	**n (%)**	**Crude RR (95% CI)**	**Adjusted RR** ^**d**^ **(95% CI)**	**n (%)**	**Crude RR (95% CI)**	**Adjusted RR** ^**e**^ **(95% CI)**
Maternal age group (years)		P = 0.001 P trend <0.001	P = 0.95 P trend = 0.86		P = 0.006 P trend = 0.001	P = 0.04 P trend = 0.009
12-19	74 (9.4)	1.48 (1.15, 1.89)	1.0 (0.76, 1.32)	24 (3.2)	1.69 (1.09, 2.62)	1.41 (0.90, 2.21)
20-35	407 (6.4)	1	1	115 (1.9)	1	1
36-50	42 (4.6)	0.73 (0.53, 1.00)	0.95 (0.67, 1.36)	8 (0.9))	0.49 (0.24, 1.00)	0.50 (0.24, 1.02)
Parity		P < 0.001; P trend < 0.001	P < 0.001; P trend < 0.001		P = 0.12; P trend = 0.04	-
0	245 (8.6)	1.66 (1.39, 1.99)	1.46 (1.20, 1.77)	62 (2.3)	1.32 (0.94, 1.84)	-
1-4	238 (5.2)	1	1	76 (1.8)	1	-
5+	18 (3.8)	0.72 (0.45, 1.17)	0.69 (0.41, 1.17)	5 (1.1)	0.62 (0.25, 1.54)	-
Type of delivery		P < 0.001	P < 0.001		P < 0.001	P < 0.001
Spontaneous vaginal delivery	461 (5.9)	1	1	127 (1.7)	1	1
Breech delivery	49 (22.5)	3.81 (2.84, 5.11)	2.44 (1.76, 3.37)	18 (8.9)	5.18 (3.16, 8.48)	3.05 (1.78, 5.20)
Vacuum extraction	14 (14.9)	2.52 (1.48, 4.30)	2.61 (1.46, 4.64)	3 (3.3)	1.91 (0.61, 6.02)	2.32 (0.74, 7.35)
Number of singleton/multiple births		P = 0.11	-		P = 0.40	-
Singleton	500 (6.4)	1	-	145 (2.0)	1	-
Multiple pregnancy (leading twins)^c^	24 (8.9)	1.40 (0.93, 2.11)	-	3 (1.2)	0.61 (0.19, 1.91)	-
Hypertensive disorder in pregnancy		P < 0.001	P = 0.12		P < 0.001	P = 0.001
No	472 (6.1)	1	1	130 (1.8)	1	1
Yes	52 (13.9)	2.28 (1.71, 3.03)	1.33 (0.93, 1.90)	18 (5.3)	2.99 (1.83, 4.90)	2.33 (1.41, 3.87)
Sex of baby		P = 0.01	P = 0.004		P = 0.16	-
Female	225 (5.7)	1	1	63 (1.7)	1	-
Male	299 (7.2)	1.25 (1.05, 1.49)	1.30 (1.09, 1.56)	85 (2.2)	1.26 (0.91, 1.75)	-
Gestation at delivery		P < 0.001	P < 0.001		P < 0.001	P < 0.001
Term	402 (5.2)	1	1	110 (1.5)	1	1
Preterm	122 (29.7)	5.70 (4.65, 6.98)	2.62 (1.94, 3.54)	38 (10.1)	6.66 (4.61, 9.64)	2.71 (1.60, 4.59)
Birth weight		P < 0.001	P < 0.001		P < 0.001	P < 0.001
Normal birth weight (≥2500 g)	349 (4.8)	1	1	94 (1.4)	1	1
Low birth weight (<2500 g)	165 (19.3)	3.99 (3.31, 4.80)	2.18 (1.67, 2.85)	52 (6.5)	4.70 (3.35, 6.59)	2.34 (1.45, 3.78)

^a^109 (1.3%) missing Apgar scores; ^b^565 (6.9%) missing Apgar scores; ^c^Only Apgar scores of leading twins were considered for the analysis; ^d^Adjusted for all other variables in the table except number of singleton/multiple births; ^e^Adjusted for maternal age, type of delivery, hypertensive disorders in pregnancy, gestation at delivery and birth weight; P-values are based on the Wald test; RR-Relative risk; CI-Confidence interval.

At 5 minutes, 148 (1.9%) of 7,669 live births whose Apgar scores were recorded had very low Apgar scores. The median Apgar score at 5 minutes was 8 (interquartile range 7–8). A significant proportion of the neonates (71%; P < 0.001, data not shown) initially with Apgar scores of 0–3 attained improved scores of 4–10 at 5 minutes. Breech delivery (aRR, 3.05; 95% CI, 1.78-5.20), vacuum extraction (aRR, 2.32; 95% CI, 0.74-7.35), preterm birth (aRR, 2.71; 95% CI, 1.60-4.59) and low birth weight (aRR, 2.34; 95% CI, 1.45-3.78) remained significantly associated with very low Apgar scores at 5 minutes. Maternal age (P trend = 0.009) and hypertensive disorders in pregnancy (aRR, 2.33; 95% CI, 1.41-3.87) which were not significantly associated with low Apgar scores in the first minute of life, became predictive of very low Apgar scores in the fifth minute, Table 3.

## Discussion

The prevalence of stillbirths and babies with very low Apgar scores are high among women with vaginal births in this study. Hypertensive disorders in pregnancy, preterm births, low birth weight, vacuum extraction and breech deliveries were not only important risk factors for stillbirth, but were also predictive of very low Apgar scores at 5 minutes. These maternal and obstetric risk factors associated with adverse vaginal birth outcomes require closer monitoring and timely interventions when necessary during delivery.

The 5.9% stillbirths observed in this study compares well with reported stillbirth rates in other tertiary hospitals in sub-Saharan Africa [5, 6, 18, 19]. In agreement with studies from these other tertiary health facilities, our stillbirth prevalence is much higher than a recent national estimate of 21 per 1000 deliveries [20]. Being a major referral centre and the only tertiary health facility in the middle sector of the country, the hospital receives high risk obstetric referrals which inevitably lead to increased adverse pregnancy outcomes, thus explaining our much higher proportion of stillbirths compared to the national estimate. Significant socio-demographic and reproductive risk factors for stillbirths in low to middle income countries include maternal age, parity, preterm delivery, low birth weight, hypertensive disorders, breech presentation as well as the sex of the baby [2, 4, 5, 7, 12, 13, 21] as was observed in this study.

Although it was not possible to distinguish between intra-uterine fetal deaths and intrapartum stillbirths in this study, unpublished reports from the centre (KATH Biostatistics Unit, 2008) indicate that about 40% of stillbirths occur during delivery, suggesting that about half of these stillbirths may not have occurred during labour. It has been suggested that up to 60% of intrapartum stillbirths can be avoided with the provision of better obstetric care including skilled attendance at birth, the use of partograph for earlier recognition of intrapartum complications (in low resource settings) and appropriate timely interventions to manage these complications [1, 21]. Despite the high turnover of vaginal deliveries in the study setting, closer monitoring of the progress of labour could possibly have saved some of these babies. A number of reasons may account for the high morbidity and mortality associated with assisted vaginal deliveries in this study. It is possible that attending staff are not strictly adhering to the departmental guidelines [15, 16] on assisted vaginal deliveries or that some of these cases may have been referred rather too late from other facilities. An assessment of the barriers to the provision of emergency obstetric care in both the referral facilities and the tertiary institution such as staff availability, training and capacities, and the availability and supervision by specialist/consultant staff, as well as timely referral patterns will be useful in improving adverse vaginal birth outcomes.

About 6.5% and 2% of live births had very low Apgar scores at 1 and 5 minutes respectively which are consistent with scores from other studies in low-resource countries [10, 22]. The obstetric factors associated with very low Apgar scores in this study have been previously reported [8, 9, 23]. It is commendable that there was a significant decrease in the proportion of babies with very low Apgar scores between the first and fifth minutes, indicating effective neonatal resuscitation in the labour wards [8]. Apgar scores in the fifth minute are generally more predictive of neonatal survival; scores of 0–3 in the fifth minute have the highest relative risk of neonatal death compared to scores of 4–6 or 7–10 [9, 22]. Although the association between very low Apgar scores in the fifth minute and neonatal deaths was not the focus of this study, neonatal mortality rate in these babies is not expected to be very different from the reported rates of 63-97% in other resource-constraint countries [10, 22]. Even in highly resourced countries such as the United States, neonatal mortality in this category could be as high as 24-31% [9].

The study has some limitations. As a retrospective analysis, the study was limited to the available data in the delivery register which excluded such factors as booking status, antepartum haemorrhage, prelabour rupture of membranes, arrival in late second stage, indication for vacuum delivery and obstetric practices such as induction and augmentation of labour. It would have been useful to categorise stillbirth into intra-uterine fetal death and intrapartum stillbirth, which is a better indicator of the quality of delivery care received. Our findings in this tertiary referral facility cannot be generalised to the rest of the population due to selection bias. Notwithstanding these limitations, the study identifies important factors associated with adverse vaginal birth outcomes in this low resource setting.

## Conclusion

The prevalence of stillbirths and very low Apgar scores associated with vaginal deliveries is high in the study setting. Although preterm birth, low birth weight and hypertensive disorders in pregnancy were significant risk factors for stillbirths and very low Apgar scores at 5 minutes, breech deliveries and vacuum extractions were the important obstetric practices associated with these adverse vaginal birth outcomes. Improving quality of obstetric care during labour (both within and outside the tertiary facility) and delivery can reduce these adverse birth outcomes. Strategies to reduce pre-labour and intrapartum stillbirths, and improve Apgar scores must be identified and strengthened.

## Abbreviations

KATH: Komfo Anokye Teaching Hospital
RR: Relative risk
CI: Confidence interval
aRR: Adjusted relative risk.
